# The association between non-high-density lipoprotein cholesterol to high-density lipoprotein cholesterol ratio with type 2 diabetes mellitus: recent findings from NHANES 2007–2018

**DOI:** 10.1186/s12944-024-02143-8

**Published:** 2024-05-21

**Authors:** Mo-Yao Tan, Li Weng, Zhong-Hao Yang, Si-Xuan Zhu, Shan Wu, Jun-Hua Su

**Affiliations:** 1 Acupuncture and Tuina School, Chengdu University of Traditional Chinese Medicine, Chengdu, Sichuan China; 2 Chengdu Integrated TCM and Western Medicine Hospital, Chengdu, Sichuan China; 3 Clinical Medical School, Chengdu University of Traditional Chinese Medicine, Chengdu, Sichuan, China

**Keywords:** NHHR, Type 2 diabetes mellitus, NHANES, Cross-sectional study

## Abstract

**Objective:**

This study aims to assess the relationship between NHHR (non-high-density lipoprotein cholesterol to high-density lipoprotein cholesterol ratio) and Type 2 diabetes mellitus (T2DM) in US adults, using National Health and Nutrition Examination Survey (NHANES) data from 2007 to 2018.

**Methods:**

This study explored the connection between NHHR and T2DM by analyzing a sample reflecting the adult population of the United States (*n* = 10,420; NHANES 2007–2018). NHHR was characterized as the ratio of non-high-density lipoprotein cholesterol to high-density lipoprotein cholesterol. T2DM was defined based on clinical guidelines. This research used multivariable logistic models to examine the connection between NHHR and T2DM. Additionally, it included subgroup and interaction analyses to assess variations among different groups. Generalized additive models, smooth curve fitting, and threshold effect analysis were also employed to analyze the data further.

**Results:**

The study included 10,420 subjects, with 2160 diagnosed with T2DM and 8260 without. The weighted multivariate logistic regression model indicated an 8% higher probability of T2DM for each unit increase in NHHR (OR: 1.08, 95% CI: 1.01–1.15) after accounting for all covariates. Subgroup analysis outcomes were uniform across various categories, demonstrating a significant positive relationship between NHHR and T2DM. Interaction tests showed that the positive link between NHHR and T2DM remained consistent regardless of age, body mass index, smoking status, moderate recreational activities, hypertension, or stroke history, with all interaction *P*-values exceeding 0.05. However, participants’ sex appeared to affect the magnitude of the connection between NHHR and T2DM (interaction *P*-value < 0.05). Also, a nonlinear association between NHHR and T2DM was discovered, featuring an inflection point at 1.50.

**Conclusions:**

Our study suggests that an increase in NHHR may be correlated with a heightened likelihood of developing T2DM. Consequently, NHHR could potentially serve as a marker for estimating the probability of T2DM development.

**Supplementary Information:**

The online version contains supplementary material available at 10.1186/s12944-024-02143-8.

## Introduction

Type 2 Diabetes Mellitus (T2DM) is a metabolic condition mainly identified by high blood sugar levels, resulting in dysfunction across multiple organs, impacting the kidneys, eyes, and cardiovascular system [1]. The hallmarks of T2DM include insufficient insulin secretion, defective insulin action, or both [2]. In 2021, around 537 million adults worldwide between 20 and 79 were estimated to have T2DM [3]. Forecasts indicate this number could rise by 46%, reaching 783 million by 2045 [3]. Developed countries, like the United States, are experiencing significant pressures on their healthcare systems due to T2DM and its associated complications [4]. Simultaneously, low-income countries are witnessing a rise in diabetes-related mortality rates, posing significant challenges to their already overstretched health systems [5].

Research has identified a strong link between dyslipidemia and T2DM [6]. High triglycerides (TG) and low-density lipoprotein cholesterol (LDL-C) levels, along with decreased high-density lipoprotein cholesterol (HDL-C), are independent risk factors for T2DM [7]. Research suggests that a higher occurrence of dyslipidemia is often a marker of inadequate T2DM management and can worsen the onset of microvascular and macrovascular complications related to T2DM [8]. Surveys conducted in several African countries indicate that non-HDL-C predominates among lipid abnormalities [9, 10]. However, research in the region suggests that LDL-C is the predominant factor [11]. Furthermore, studies show that the occurrence of dyslipidemia in T2DM patients can be as high as 94%, but in developed countries such as the USA and the UK, the prevalence is comparatively lower [12, 13]. This highlights the limitations and lack of uniformity in using traditional lipid profiles to assess T2DM, urgently calling for the establishment of new lipid assessment index to explore their relationship with T2DM further.

The ratio of non-HDL-C to HDL-C (NHHR) is increasingly regarded as an innovative and comprehensive marker for evaluating atherosclerosis lipid composition [14]. Research has shown that NHHR not only effectively assesses atherosclerosis severity but also closely correlates with its relevance and predictive value for various diseases. For instance, studies by Wen et al. have demonstrated an association between NHHR and chronic kidney disease, revealing a negative correlation with the estimated glomerular filtration rate [15]. Additionally, research focusing on Asian populations has highlighted that NHHR is associated with the regulation of non-alcoholic fatty liver disease, noting a 64.5% increase in alanine aminotransferase/aspartate aminotransferase levels for every unit increase in the NHHR [16, 17]. Further research by Kim SW has confirmed that NHHR has a higher diagnostic value for predicting insulin resistance (IR) and metabolic syndrome compared to traditional lipid markers [18]. These studies collectively suggest that NHHR may serve as an effective tool for predicting metabolic-related diseases. Therefore, investigating the link between NHHR and T2DM holds significant scientific value, positioning NHHR as a novel marker that could pave new pathways for assessing the likelihood of and managing T2DM.

Therefore, we conducted a comprehensive analysis to explore the relationship between NHHR and T2DM in U.S. adults, utilizing national health and nutrition examination surveys (NHANES) data from 2007 to 2018, with the goal of offering significant epidemiological insights.

## Methods

### Study population

The NHANES database, operated by the Centers for Disease Control and Prevention, conducts multi-stage surveys to provide comprehensive insights into the health and nutritional status of the U.S. population. The data collection method uses a layered strategy, consistently collecting data biennially. The NHANES was authorized by the Ethics Review Committee of the National Health Statistics Research Center, and all participants provided their written, informed consent [19]. Comprehensive details can be found on their website: https://www.cdc.gov/nchs/nhanes/. This study was meticulously conducted in accordance with the Strengthening the Reporting of Observational Studies in Epidemiology (STROBE) guidelines (Supplementary Material 1) [20].

**Fig. 1:**
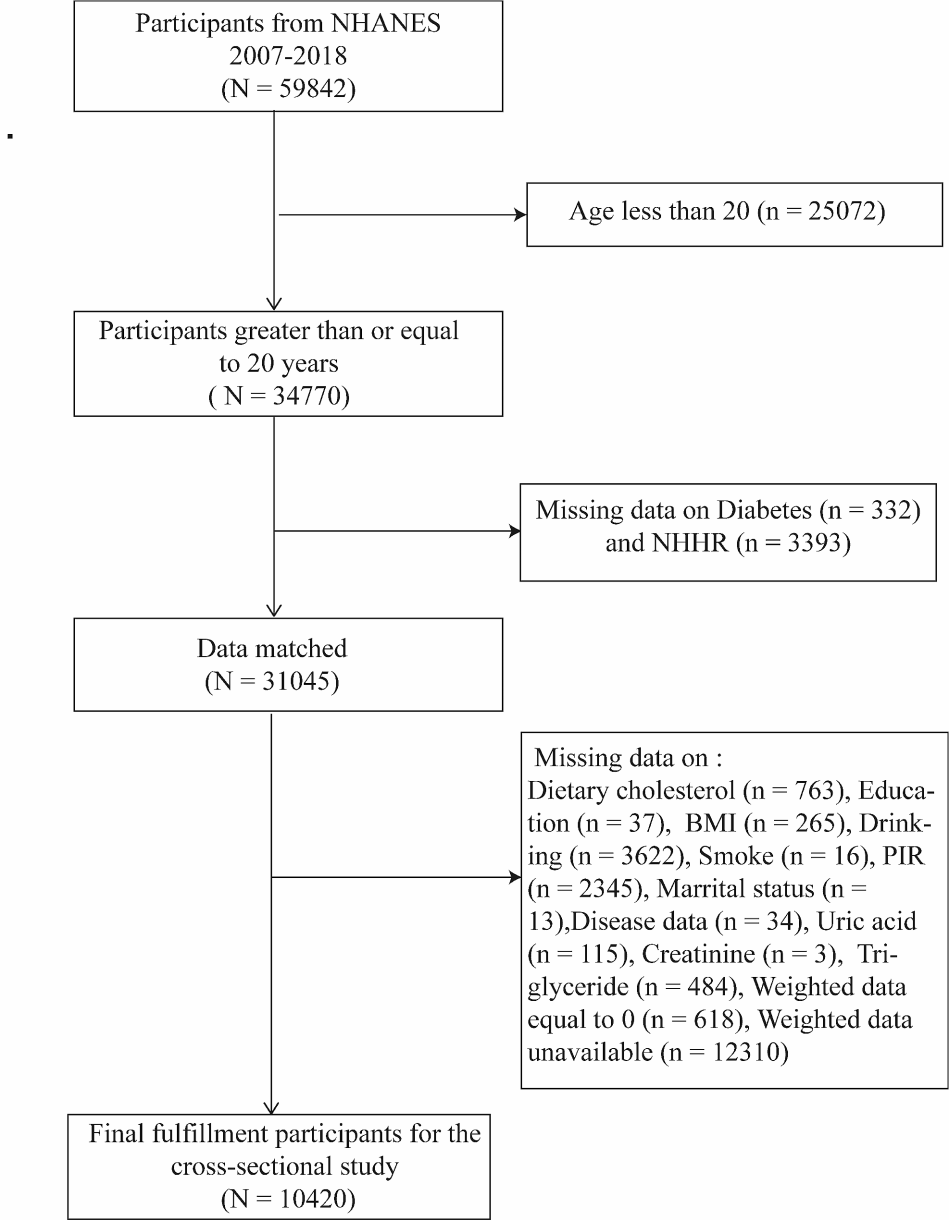
Flowchart of the sample selection from the 2007–2018 National Health and Nutrition Examination Survey (NHANES).

This cross-sectional research examined data from individuals over six consecutive NHANES cycles, spanning from 2007 to 2018. Initially, 59,842 participants were included. To identify the final study population, we applied the following exclusion criteria: (1) Participants younger than 20 years old; (2) Missing data on NHHR and T2DM; (3) Absence of data on covariates such as dietary cholesterol intake, education level, body mass index (BMI), alcohol consumption, smoking, poverty income ratio (PIR), marital status, hypertension, stroke, serum uric acid, TG and creatinine; (4) Participants missing weight data and those with a recorded weight of 0. After these exclusions, the study ultimately comprised 10,420 subjects, as depicted in Fig. 1.

### Exposure variable

In this study, NHHR was utilized as the exposure variable. As described in previous literature [14, 21], NHHR represents the ratio of non-HDL-C to HDL-C. Non- HDL-C is determined by subtracting HDL-C from the total cholesterol (TC) level.

### Outcome variable

T2DM diagnosis was established through one or more of the following criteria [22]: (1) physician-confirmed clinical diagnosis, (2) hemoglobin A1c (HbA1c) percentage of 6.5% or higher, (3) fasting glucose level of 7.0 mmol/L or more, (4) random blood glucose measurement of 11.1 mmol/L or higher, (5) blood glucose level of at least 11.1 mmol/L during a 2-hour oral glucose tolerance test (OGTT), or (6) current use of T2DM management medications or insulin.

### Covariates

Drawing from prior studies [14, 21], we incorporated variables potentially impacting the link between NHHR and T2DM. Included covariates encompass age, sex, race, PIR, marital status, BMI, waist circumference, education level, smoking, alcohol consumption, creatinine, moderate recreational activities, blood urea nitrogen, serum uric acid, dietary cholesterol on the first day, stroke, and hypertension. Comprehensive descriptions of the covariates are provided in Supplementary Material 2.

### Statistical analysis

Every statistical analysis includes the sampling weights, stratification, and clustering from the NHANES study to accurately mirror the intricate multi-stage sampling strategy, ensuring the sample effectively represents the non-institutionalized U.S. population. This approach ensures precise estimates without overstating statistical significance. In choosing weights, the official NHANES guidelines recommend first determining the variable that represents the smallest population group, followed by selecting the corresponding weight for that variable. In our study, the data encompasses results from the mobile examination center, including TC and HDL-C data. Following the recommendations of the weight selection guidelines, we opted for the specific sub-weight (WTSAF2YR) corresponding to TC and HDL-C. For the merged survey cycles, a new sampling weight was created by dividing each cycle’s two-year weight by six in accordance with the NHANES analysis guide [23]. Continuous variables were presented as mean values with their standard errors, and categorical variables were reported in percentages. To evaluate differences between groups, weighted Student’s t-tests were used for continuous variables, and weighted chi-square tests were applied to categorical variables. In order to enhance the precision of the results, factors that may introduce bias were considered. Additionally, the association between NHHR and T2DM was investigated using three distinct weighted logistic regression models.

Model 1 did not adjust for any additional variables. In contrast, Model 2 adjusted for basic demographic factors such as gender, age, and race. Model 3 included a comprehensive set of adjustments covering gender, age, race, moderate recreational activities, levels of creatinine and blood urea nitrogen, serum uric acid, dietary cholesterol intake, education, PIR, marital status, BMI, waist circumference, alcohol use, smoking, hypertension, and stroke. Moreover, NHHR, as a continuous variable, was divided into tertiles for a more nuanced analysis. Specifically, the divisions were as follows: the T1 tertile (lowest third) included NHHR of 2.107 or less, the T2 tertile (middle third) encompassed NHHR between 2.107 and 3.134, and the T3 tertile (highest third) consisted of NHHR exceeding 3.134. Extensive analyses were conducted to examine the effects within subgroups defined by age (≤ 65 years or > 65 years), gender (female or male), BMI categories (underweight, normal, overweight, or obese), smoking status (never, former, or current smokers), stroke history (yes or no), hypertension status (yes or no), and level of moderate recreational activity (inactive or active). Interaction tests were employed to investigate the heterogeneity in the association between NHHR and T2DM within various subgroups. In order to delve deeper into the nonlinear nature of the NHHR and T2DM relationship, methods such as generalized additive models (GAM), smooth curve fitting, and threshold effect analysis were utilized. The study’s statistical relevance was confirmed using a two-sided P-value threshold of 0.05. The analysis was performed utilizing R version 4.3.2 and the Empower software, which can be accessed at www.empowerstats.com.

## Results

### Participants characteristics

Table 1 presents the baseline characteristics of the participants stratified by their T2DM status, with 2160 individuals in the T2DM group and 8260 individuals in the non-T2DM group. The T2DM group had an average age of 59.03 ± 0.40 years, consisting of 50.53% males and 49.47% females. Their NHHR value was 2.99 ± 0.04, and their dietary cholesterol intake was 296.89 ± 6.78 mg. On the other hand, the non-T2DM group’s average age was 44.93 ± 0.31 years, with a gender distribution of 49.39% males and 50.61% females. The NHHR for this group stood at 2.73 ± 0.02, with a dietary cholesterol intake of 297.62 ± 3.33 mg. Patients with T2DM have significantly elevated levels of age, BMI, waist circumference, TG, insulin, fasting glucose, HbA1c, blood urea nitrogen, serum uric acid, creatinine, and NHHR compared to those in the non-T2DM group, with all differences reaching statistical significance (*P* < 0.05).

**Table 1:** Weighted baseline characteristics of participants

	Non-T2DM (*n* = 8260)	T2DM (*n* = 2160)	*P*-value
**Sociodemographic**			
**Age; years, Mean (SE)**	44.93 (0.31)	59.03 (0.40)	**< 0.001**
**Sex (%)**			0.47
Female	50.61	49.47	
Male	49.39	50.53	
**Race (%)**			**< 0.001**
Mexican American	7.82	9.16	
Non-Hispanic White	69.97	65.58	
Non-Hispanic Black	9.99	13.08	
Other Race	12.22	12.19	
**Education Level (%)**			**< 0.001**
Less Than 9th Grade	3.79	8.23	
9-11th Grade (Includes 12th grade with no diploma)	9.74	13.09	
College Graduate or above	33.05	21.63	
High School Grad/GED or Equivalent	21.94	26.48	
Some college or AA degree	31.48	30.57	
**Ratio of family income to poverty (%)**			**< 0.001**
low-income	13.72	14.60	
middle-income	47.90	55.31	
high-income	38.38	30.09	
**Marital Status (%)**			**< 0.001**
Divorced	9.97	12.30	
Living with partner	8.97	4.55	
Married	54.84	61.49	
Never married	19.90	9.28	
Separated	2.08	2.32	
Widowed	4.23	10.06	
**Anthropometric**			
**Body Mass Index; kg/m**^**2**^, **Mean (SE)**	28.31 (0.12)	32.73 (0.21)	**< 0.001**
**Waist circumference; cm, Mean (SE)**	97.29 (0.29)	110.63 (0.50)	**< 0.001**
**Healthy behavior factors**			
**Dietary cholesterol intake; mg, Mean (SE)**	297.62 (3.33)	296.89 (6.78)	0.92
**Smoking status (%)**			**< 0.001**
Never	56.63	50.77	
Former	23.84	33.68	
Now	19.53	15.55	
**Alcohol intake (%)**			**< 0.001**
Never	9.33	14.56	
Former	10.84	20.28	
Mild	38.71	37.80	
Moderate	18.60	13.32	
Heavy	22.52	14.04	
**Moderate recreational activities (%)**			**< 0.001**
Inactive	42.41	60.44	
Active	57.59	39.56	
**Biochemical data**			
**Total cholesterol; mmol/L, Mean (SE)**	4.99 (0.02)	4.77 (0.04)	**< 0.001**
**Blood Urea Nitrogen; mmol/L, Mean (SE)**	4.70 (0.03)	5.65 (0.06)	**< 0.001**
**Serum uric acid; µmol/L, Mean (SE)**	322.95 (1.33)	348.35 (2.70)	**< 0.001**
**Creatinine; µmol/L, Mean (SE)**	76.47 (0.34)	85.23 (1.53)	**< 0.001**
**HDL-C; mmol/L, Mean (SE)**	1.43 (0.01)	1.28 (0.01)	**< 0.001**
**LDL-C; mmol/L, Mean (SE)**	2.99 (0.01)	2.76 (0.03)	**< 0.001**
**TG; mmol/L, Mean (SE)**	1.25 (0.01)	1.62 (0.03)	**< 0.001**
**Insulin; µU/mL, Mean (SE)**	11.25 (0.17)	20.15 (0.65)	**< 0.001**
**Fasting glucose; mmol/L, Mean (SE)**	5.48 (0.01)	8.10 (0.09)	**< 0.001**
**HOMA-IR; units, Mean (SE)**	2.80 (0.05)	7.59 (0.30)	**< 0.001**
**HbA1c; %, Mean (SE)**	5.37 (0.01)	6.91 (0.05)	**< 0.001**
**NHHR; units, Mean (SE)**	2.73 (0.02)	2.99 (0.04)	**< 0.001**
**Chronic disease factors**			
**Hypertension (%)**			**< 0.001**
No	68.46	31.20	
Yes	31.54	68.80	
**Stroke (%)**			**< 0.001**
No	97.73	93.49	
Yes	2.27	6.51	

**Notes**: All values are presented as proportion (%) for categorical variables, assessed via weighted chi-square tests, or mean (standard error) for continuous variables, assessed via weighted Student’s t-tests

**Abbreviations**: **NHHR**: non-high-density lipoprotein cholesterol to high-density lipoprotein cholesterol ratio; **T2DM**: type II diabetes mellitus; **TG**: Triglyceride; **HDL-C**: high-density lipoprotein cholesterol; **LDL-C**: low-density lipoprotein cholesterol; **HOMA-IR**: Homeostatic Model Assessment of Insulin Resistance; **HbA1c**: hemoglobin A1c; **SE**: standard error

Supplementary Material 3 offers a comprehensive review of the distribution and characteristics of study subjects across various NHANES cycles. The distribution of socio-demographic characteristics and chronic diseases (e.g., hypertension, stroke, T2DM), among the participants showed no significant differences over time (all *P* > 0.05). However, levels of NHHR decreased yearly (*P* < 0.001). In the 2017–2018 period, participants had higher values of blood urea nitrogen and lower values of TC, LDL-C, and TG compared to the previous survey period (all *P* < 0.05).

### The association between NHHR with T2DM

Table 2 demonstrates a statistically significant positive correlation between NHHR and T2DM in three models: a model without covariates (model 1: OR = 1.17; 95% CI, 1.12–1.23), a model adjusted for sex, age, and race (model 2: OR = 1.25; 95% CI, 1.19–1.33), and a model adjusted for all covariates (model 3: OR = 1.08; 95% CI, 1.01–1.15). This positive association remains consistent across all models. To further explore the relationship between NHHR and T2DM, we divided NHHR into three tertiles. In Model 2, when compared to the lowest tertile of NHHR (Tertile 1), the odds of having T2DM were 92% higher in the highest tertile (OR = 1.92; 95% CI: 1.59–2.31). However, after applying a more comprehensive adjustment, the association observed (OR = 1.13; 95% CI: 0.90–1.42) was not statistically significant.

**Table 2:** Weighted Multivariate logistic regression models of NHHR with T2DM.

NHHR	OR^a^ (95% CI), *P*-value		
	Model 1^b^	Model 2^c^	Model 3^d^
**Continuous**	1.17 (1.12,1.23) < 0.001	1.25 (1.19,1.33) < 0.001	1.08 (1.01,1.15) 0.02
**Categories**			
**Tertile 1** (< 2.107)	**Reference**	**Reference**	**Reference**
**Tertile 2** (2.107–3.134)	1.35 (1.12,1.63) 0.002	1.40 (1.15,1.71) < 0.001	1.00 (0.81,1.22) 0.96
**Tertile 3** (> 3.134)	1.64 (1.40,1.92) < 0.001	1.92 (1.59,2.31) < 0.001	1.13 (0.90,1.42) 0.28

**Notes**: In sensitivity analysis, NHHR is transformed from a continuous variable to a categorical variable (Tertiles); **OR**^**a**^: effect size; **Model 1**^**b**^: no covariates were adjusted; **Model 2**^**c**^: adjusted for sex, age, and race; **Model 3**^**d**^: adjusted for sex, age, race, moderate recreational activities, creatinine, blood urea nitrogen, serum uric acid, dietary cholesterol intake, education level, ratio of family income to poverty, marital status, body mass index, waist circumference, alcohol intake, smoking status, hypertension and stroke; NHHR, as a continuous variable, was divided into tertiles for a more nuanced analysis. Specifically, the divisions were as follows: the T1 tertile (lowest third) included NHHR of 2.107 or less, the T2 tertile (middle third) encompassed NHHR between 2.107 and 3.134, and the T3 tertile (highest third) consisted of NHHR exceeding 3.134

**Abbreviations**: **NHHR**: non-high-density lipoprotein cholesterol to high-density lipoprotein cholesterol ratio; **T2DM**: type II diabetes mellitus; **95% CI**: 95% confidence interval; **OR**: odds ratio

### Subgroup analysis

Subgroup analyses and interaction tests were conducted to examine the influence of population stratification variables on the association between NHHR and T2DM, as outlined in Table 3. The positive associations between NHHR and T2DM consistently persisted within distinct subgroups. Notably, there were no significant interactions observed among age, BMI, smoking status, hypertension, stroke, and moderate recreational activities, suggesting that the positive association was not influenced by these variables (all *P*-values for interaction > 0.05). However, gender significantly influenced the relationship between NHHR and T2DM, with an interaction *P*-value < 0.05. Analysis showed that females had a higher likelihood than males (OR, 1.16 [95% CI, 1.05–1.29]) of developing T2DM, indicating gender differences in how NHHR relates to the likelihood of T2DM, with females showing a more pronounced connection.

**Table 3:** Subgroup analysis for the association between NHHR and T2DM.

	OR (95%CI)	*P*-value	*P* for interaction Interaction coefficient
**Age (years)**			0.33
<65	1.24 (1.17–1.31)	<0.001	0.09
≥ 65	1.05 (0.93–1.18)	0.41	0.01
**Sex**			< 0.001
Male	1.16 (1.09–1.24)	<0.001	0.05
Female	1.27 (1.17–1.38)	<0.001	0.12
**BMI((kg/m** ^**2**^ **)**			0.59
underweight or normal or overweight (< 30 kg/m^2^)	1.14 (1.05–1.23)	0.002	0.04
Obese (≥ 30 kg/m^2^)	1.14 (1.07–1.21)	<0.001	0.10
**Smoking status**			0.14
Never	1.26 (1.17–1.36)	<0.001	0.08
Former	1.14 (1.04–1.25)	0.01	0.07
Now	1.15 (1.05–1.26)	0.01	0.09
**Stroke**			0.54
No	1.21 (1.14–1.27)	<0.001	0.08
Yes	1.11 (0.95–1.31)	0.19	0.08
**Hypertension**			0.49
No	1.15 (1.05, 1.26)	0.003	0.02
Yes	1.11 (1.03, 1.18)	0.004	0.15
**Moderate recreational activities**			0.06
Inactive	1.13 (1.06, 1.21)	<0.001	0.09
Active	1.26 (1.18, 1.35)	<0.001	0.06

Note: The subgroup analyses shown in the table were conducted based on Model 3, with the inclusion of covariates including: sex, age, race, moderate recreational activities, creatinine, blood urea nitrogen, serum uric acid, dietary cholesterol intake, education level, ratio of family income to poverty, marital status, body mass index, waist circumference, alcohol intake, smoking status, hypertension and stroke

**Abbreviation**: **BMI**: body mass index; **T2DM**: type II diabetes mellitus; **95% CI**: 95% confidence interval; **OR**: odds ratio

### Nonlinear relationships between NHHR and T2DM

**Fig. 2:**
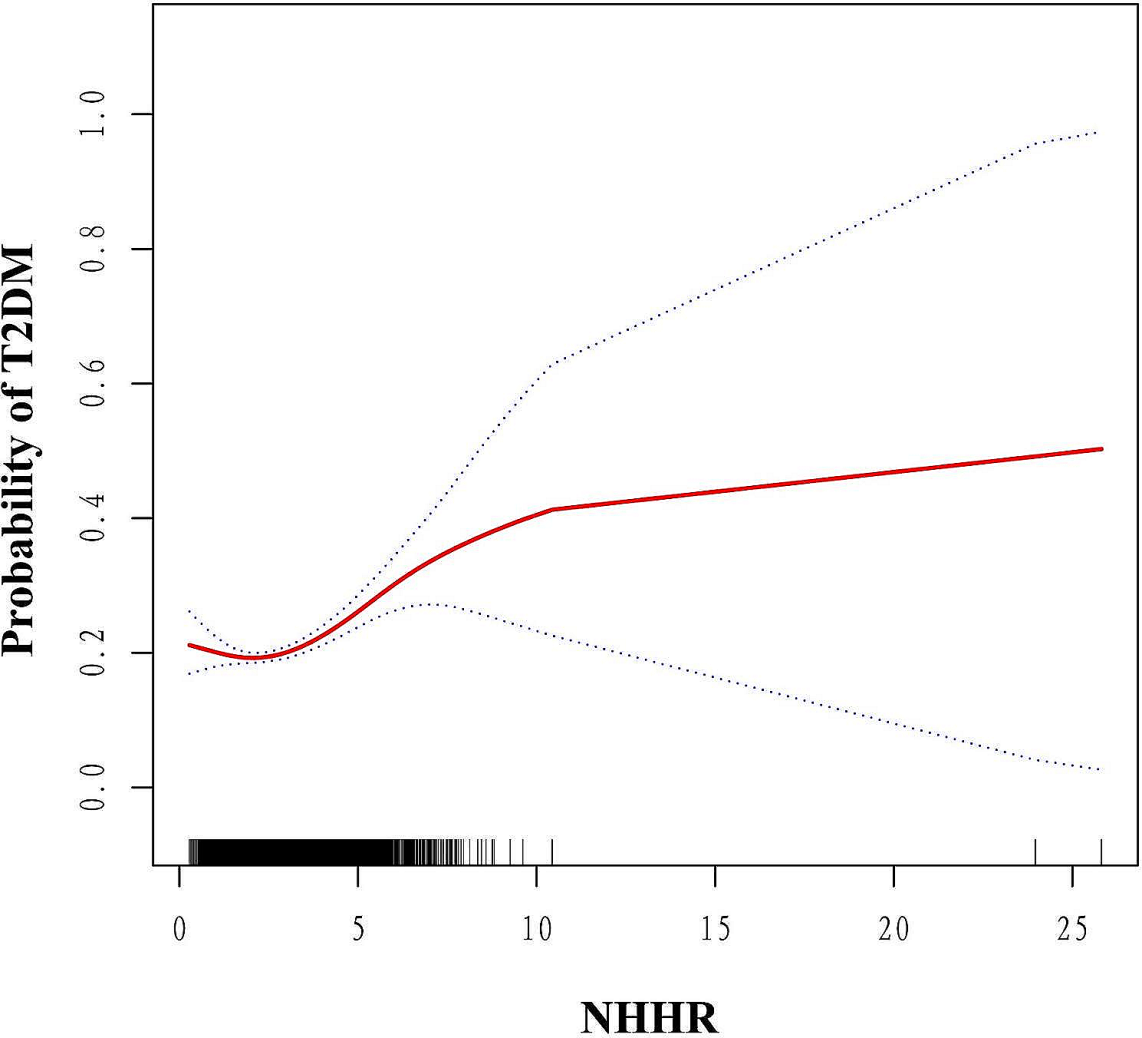
smoothed curve fitting: Dose-response relationship between NHHR and T2DM.

Figure 2 presents the results of the GAM along with its smoothing curve fitting, uncovering a nonlinear relationship between NHHR and T2DM. The log-likelihood ratio test revealed a statistically significant difference between the linear and the piecewise linear regression models, evidenced by a P-value of less than 0.017. Table 4 identifies an inflection point in the NHHR-T2DM relationship at an NHHR of 1.50. Below this threshold, the OR was 0.70 (95% CI: 0.40, 1.10), indicating a non-significant association. On the contrary, exceeding this threshold revealed a significant and positive link between NHHR and T2DM, evidenced by an OR of 1.20 (95% CI: 1.17 to 1.30), highlighting the notable positive connection between increased NHHR levels and T2DM.

**Table 4:** Threshold effect analysis of NHHR on T2DM using two-piecewise linear regression model

NHHR	Adjust OR (95% CI) *P* value
**Fitting by linear regression model**	1.20 (1.10, 1.20) < 0.001
**Fitting by two-piecewise linear regression model**	
**Inflection point**	1.50
< 1.50	0.70 (0.40, 1.10) 0.09
> 1.50	1.20 (1.17, 1.30) < 0.001
**Log likelihood ratio test**	0.017

Note: Adjusted for sex, age, race, moderate recreational activities, creatinine, blood urea nitrogen, serum uric acid, dietary cholesterol intake, education level, ratio of family income to poverty, marital status, body mass index, waist circumference, alcohol intake, smoking status, hypertension and stroke

**Abbreviations**: **NHHR**: non-high-density lipoprotein cholesterol to high-density lipoprotein cholesterol ratio; **T2DM**: type II diabetes mellitus; **OR**: odds ratio; **95% CI**: 95% confidence interval

## Discussion

### Summary of results

In this cross-sectional analysis of 10,420 American participants, the findings suggest that those with elevated NHHR may potentially face a greater likelihood of developing T2DM. Subgroup analyses further confirmed the stability of this positive correlation. Importantly, this correlation was not affected by age, BMI, smoking status, hypertension, stroke, or moderate recreational activities. Specifically, gender could significantly impact the intensity of the link between NHHR and T2DM susceptibility. With the same rise in NHHR, women are more likely to develop T2DM than men. Furthermore, we discovered a nonlinear relationship between NHHR and T2DM, with a turning point identified at an NHHR of 1.50. The NHHR serves as an early indicator of the likelihood of developing T2DM, holding significant value for early prevention and diagnosis in high-risk populations [24]. Research shows a strong correlation between NHHR and the likelihood of developing T2DM [25], which is capable of predicting elevated fasting blood glucose levels and thereby providing a foundation for early preventative measures [26]. Furthermore, for patients diagnosed with T2DM, NHHR aids physicians in assessing complications and prognoses, optimizing treatment plans, and enhancing therapeutic outcomes [27]. Its application extends beyond individual patient management to influencing public health policies and promoting healthy lifestyles.

### The correlation between NHHR and the increased likelihood of T2DM

Our research suggests that NHHR could be a contributing factor to the development of T2DM, consistent with previous findings. Firstly, research conducted in Japan has identified NHHR as an independent and stable predictor for the future likelihood of developing T2DM, demonstrating its superior precision in assessing the probability of T2DM when compared to conventional lipid markers [25]. Additionally, a cohort study from China corroborates the positive correlation between NHHR and the incidence of T2DM, thereby affirming the link between elevated NHHR levels and an augmented risk of developing T2DM [24]. Worth noting is the extensive exploration of the connection between T2DM and dyslipidemia. The NHHR, a novel composite index, reflects the lipid composition associated with atherosclerosis, which in turn increases the likelihood of T2DM complications [24, 25]. Numerous studies have identified a tight association between high levels of Non-HDL-C and low levels of HDL-C with the increased likelihood of developing T2DM [28, 29]. In a sweeping analysis involving more than three million individuals, a U-shaped relationship between HDL-C levels and clinical outcomes in T2DM patients was observed [30]. It is particularly noted that an increment in HDL-C levels below 60 mg/dl is associated with a decreased risk of T2DM and its complications [30]. Furthermore, IR, which is associated with abnormal lipid values, emerges as a critical factor influencing the likelihood of T2DM development [31]. Animal studies have further substantiated that diminishing adipose tissue can notably ameliorate T2DM conditions in rodents [32]. This includes mitigating the accumulation of visceral fat, fostering the breakdown of fats, and enhancing the oxidation of fatty acids [33]. During these processes, genetic transcriptional regulation within adipose tissue is instrumental in reducing HDL-C levels and elevating TG levels [34]. Simultaneously, these research outcomes, in conjunction with our findings, solidify the evidence of a nexus between NHHR and T2DM, underscoring the significance of NHHR as a predictor and its potential role in assessing and managing the likelihood of developing T2DM.

### The mechanisms underlying the Positive Association between NHHR and T2DM

The mechanistic link between NHHR and T2DM remains unclear, with several hypotheses currently proposed: First, increased NHHR levels may indicate a higher risk of cholesterol deposition in peripheral tissues, leading to accelerated movement of cholesterol from the bloodstream to peripheral cells [35]. This increase can elevate free fatty acid (FFA) concentrations, reducing insulin sensitivity [36]. Additionally, sustained high levels of FFAs might suppress AMP-activated protein kinase activity and encourage TG accumulation [37]. This not only keeps TG levels high but could also cause IR or pancreatic beta-cell dysfunction. Second, cholesterol buildup in peripheral cells may boost the activity of peroxisome proliferator-activated receptor gamma, increasing peripheral IR [38]. At the same time, lipid-induced oxidative stress could activate protein tyrosine phosphatase 1B and SH2-containing protein tyrosine phosphatases, disrupting essential elements of the insulin signaling pathway, thus terminating insulin activity and increasing the likelihood of T2DM development [39]. Furthermore, cholesterol accumulation in the pancreas may deteriorate islet cell function [40]. Cholesterol could lead to the clustering of islet amyloid polypeptide, resulting in islet amyloid formation that worsens beta-cell dysfunction and disrupts glucose management [27]. Alternatively, cholesterol may activate transcription factor EB, affecting autophagy and causing beta-cell apoptosis [41]. Lastly, a rise in NHHR suggests a decrease in HDL-C levels. Studies indicate that HDL-C has anti-diabetic effects, such as enhancing insulin sensitivity, aiding glucose regulation, preserving beta-cell integrity, preventing non-HDL-C oxidation, and mitigating inflammation [6, 42]. Thus, an increased NHHR could compromise HDL-C’s protective role on islet cells, potentially heightening the likelihood of T2DM development.

### Analyzing the outcomes of Subgroup and Interaction effects

Our subgroup and interaction analysis revealed a significant finding: despite similar increases in NHHR, females are at a higher probability of developing T2DM than males, a result supported by prior research. For example, a cohort study in Japan demonstrated that females with dyslipidemia are more likely to develop T2DM compared to males [43]. Furthermore, a study that included a significant number of African American and Hispanic American participants without T2DM found that females had higher TG/HDL-C levels, which are 80% sensitive in predicting T2DM [44]. Similarly, a case-control study of 1,142 Singaporean Chinese individuals discovered a strong correlation between increased TG/HDL-C ratios and a significantly higher risk of T2DM in females [45]. Research by Cheng et al., involving 4173 Chinese males and 6568 females, further confirmed the TG/HDL-C ratio as an independent risk factor for T2DM, especially among females, aligning with our study’s findings [46]. The underlying mechanisms remain partly unclear but are thought to involve several key factors. Firstly, estrogen in females generally benefits cardiovascular health and glucose metabolism [47]. However, these protective effects diminish with age, particularly after menopause, exacerbating the adverse outcomes of elevated NHHR [48]. Secondly, women are more prone to accumulate fat in the abdomen and hips, leading to increased systemic inflammation [49]. Recent studies suggest that inflammatory markers produced by adipose tissue contribute to T2DM risk factors [50].

### Strength and limitation

This study showcases several advantages. Initially, based on the analysis of a substantial sample size of 10,420 participants, this study first and foremost provides epidemiological evidence of the relationship between NHHR and T2DM in the American population. Additionally, the data utilized originates from the NHANES database, encompassing a representative sample of individuals from diverse regions throughout the United States. Through the application of suitable sampling weights, the findings of this study can be effectively extrapolated to the entirety of the United States population. Lastly, by building upon preliminary research, this study has meticulously accounted for various confounding variables, effectively reducing the potential biases that these factors might introduce.

However, acknowledging certain limitations is crucial. Initially, given the study’s cross-sectional design, it is not possible to establish a causal relationship between NHHR and T2DM. Secondly, a limitation of our study is the cross-sectional design itself, which constrains our capacity to exclude the possibility of an inverse association between NHHR and the development of T2DM. Additionally, despite adjustments for several potential confounders, the presence of unmeasured variables could still influence the outcomes. Finally, in light of the study’s emphasis on the United States demographic, prudence is recommended when extrapolating the results to different populations.

## Conclusions

Our results suggest a probability of a positive correlation between NHHR and T2DM in U.S. adults. Future investigations will require more high-quality studies to confirm our findings.

### Electronic supplementary material

Below is the link to the electronic supplementary material.

Supplementary Material 1

## Data Availability

No datasets were generated or analysed during the current study.

## Abbreviations

NHHR: non-high-density lipoprotein cholesterol to high-density lipoprotein cholesterol ratio
T2DM: Type 2 diabetes mellitus
TG: Triglyceride
TC: Total cholesterol
HDL-C: high-density lipoprotein cholesterol
LDL-C: low-density lipoprotein cholesterol
NHANES: National Health and Nutrition Examination Survey
FFA: free fatty acid
BMI: body mass index
PIR: poverty income ratio
OR: Odds ratio
CI: Confidence interval
IR: insulin resistance
STROBE: Strengthening the Reporting of Observational Studies in Epidemiology
