# Supplementary material for: The association between non-high-density lipoprotein cholesterol to high-density lipoprotein cholesterol ratio with type 2 diabetes mellitus: recent findings from NHANES 2007–2018
Source: Lipids Health Dis. 2024 May 21;23:151. doi: 10.1186/s12944-024-02143-8 (PMC11106869; doi:10.1186/s12944-024-02143-8)
Supplement: Supplementary file 1 — Supplementary Material 1 [file 12944_2024_2143_MOESM1_ESM.docx]

**Supplementary Material 1. STROBE Statement—Checklist of items that should be included in reports of *cross-sectional studies.***

|  | **Item No** | **Recommendation** | **Page No** |
| --- | --- | --- | --- |
| **Title and abstract** | 1 | (*a*) Indicate the study’s design with a commonly used term in the title or the abstract | 1 |
|  |  | (*b*) Provide in the abstract an informative and balanced summary of what was done and what was found | 2-3 |
| **Introduction** | | | |
| Background/rationale | 2 | Explain the scientific background and rationale for the investigation being reported | 4-6 |
| Objectives | 3 | State specific objectives, including any prespecified hypotheses | 7 |
| **Methods** | | | |
| Study design | 4 | Present key elements of study design early in the paper | 7-8 |
| Setting | 5 | Describe the setting, locations, and relevant dates, including periods of recruitment, exposure, follow-up, and data collection | 7-8 |
| Participants | 6 | (*a*) Give the eligibility criteria, and the sources and methods of selection of participants | 8 |
| Variables | 7 | Clearly define all outcomes, exposures, predictors, potential confounders, and effect modifiers. Give diagnostic criteria, if applicable | 8-11 |
| Data sources/ measurement | 8* | For each variable of interest, give sources of data and details of methods of assessment (measurement). Describe comparability of assessment methods if there is more than one group | *7-8* |
| Bias | 9 | Describe any efforts to address potential sources of bias | 7-8 |
| Study size | 10 | Explain how the study size was arrived at | 8 |
| Quantitative variables | 11 | Explain how quantitative variables were handled in the analyses. If applicable, describe which groupings were chosen and why | 11-12 |
| Statistical methods | 12 | (*a*) Describe all statistical methods, including those used to control for confounding | 11-12 |
|  |  | (*b*) Describe any methods used to examine subgroups and interactions | 11-12 |
|  |  | (*c*) Explain how missing data were addressed | 8 |
|  |  | (*d*) If applicable, describe analytical methods taking account of sampling strategy | 11-12 |
|  |  | (*e*) Describe any sensitivity analyses | 15 |
| **Results** | | | |
| Participants | 13* | (a) Report numbers of individuals at each stage of study—eg numbers potentially eligible, examined for eligibility, confirmed eligible, included in the study, completing follow-up, and analysed | 8 |
|  |  | (b) Give reasons for non-participation at each stage | / |
|  |  | (c) Consider use of a flow diagram | Fig 1 |
| Descriptive data | 14* | (a) Give characteristics of study participants (eg demographic, clinical, social) and information on exposures and potential confounders | 8 |
|  |  | (b) Indicate number of participants with missing data for each variable of interest | 8 |
| Outcome data | 15* | Report numbers of outcome events or summary measures | 5 |
| Main results | 16 | (*a*) Give unadjusted estimates and, if applicable, confounder-adjusted estimates and their precision (eg, 95% confidence interval). Make clear which confounders were adjusted for and why they were included | 12-15 |
|  |  | (*b*) Report category boundaries when continuous variables were categorized | 12-15 |
|  |  | (*c*) If relevant, consider translating estimates of relative risk into absolute risk for a meaningful time period | / |
| Other analyses | 17 | Report other analyses done—eg analyses of subgroups and interactions, and sensitivity analyses | 15-16 |
| **Discussion** | | | |
| Key results | 18 | Summarise key results with reference to study objectives | 18 |
| Limitations | 19 | Discuss limitations of the study, taking into account sources of potential bias or imprecision. Discuss both direction and magnitude of any potential bias | 18 |
| Interpretation | 20 | Give a cautious overall interpretation of results considering objectives, limitations, multiplicity of analyses, results from similar studies, and other relevant evidence | 16-18 |
| Generalisability | 21 | Discuss the generalisability (external validity) of the study results | 16-18 |
| **Other information** | | | |
| Funding | 22 | Give the source of funding and the role of the funders for the present study and, if applicable, for the original study on which the present article is based | / |

*Give information separately for exposed and unexposed groups.

**Notes:** An Explanation and Elaboration article discusses each checklist item and gives methodological background and published examples of transparent reporting. The STROBE checklist is best used in conjunction with this article (freely available on the Web sites of PLoS Medicine at http://www.plosmedicine.org/, Annals of Internal Medicine at http://www.annals.org/, and Epidemiology at http://www.epidem.com/). Information on the STROBE Initiative is available at [www.strobe-statement.org.](http://www.strobe-statement.org.)

**Supplementary Material 2. Detailed information on covariates.**

| **Covariate** | **Range** | **Grouping for adjustment** |
| --- | --- | --- |
| **Race** | **NA** | Mexican American |
|  |  | Non-Hispanic white |
|  |  | Non-Hispanic black |
|  |  | Other races |
|  |  |  |
| **Education level** | **NA** | Less Than 9th Grade |
|  |  | 9-11th Grade (Includes 12th grade with no diploma) |
|  |  | College Graduate or above |
|  |  | High School Grad/GED or Equivalent |
|  |  | Some college or AA degree |
|  |  |  |
| **Income level [1]** | **PIR: 0.00-5.00 ("5.00" means ≥ 5.00)** | low-income PIR: (0.00-1.00] |
|  |  | middle-income PIR: (1.00-4.00) |
|  |  | high-income PIR: [4.00-5.00] |
|  |  |  |
| **Smoking status** | **NA** | Yes |
|  |  | No |
|  |  |  |
| **Alcohol consumption [2]** | **NA** | had <12 drinks in lifetime |
|  |  | had ≥12 drinks in 1 year and did not drink last year, or did not drink last year but drank ≥12 drinks in lifetime |
|  |  | 1 is for female and 2 is for male |
|  |  | 2 is for female and 3 is for male |
|  |  | 3 is for female and 4 is for male |
|  |  |  |
| **Diabetes [3]** | **NA** | 1.doctor told you have diabetes |
|  |  | 2. glycohemoglobin HbA1c(%) >= 6.5 |
|  |  | 3. fasting glucose (mmol/l) >= 7.0 |
|  |  | 4. random blood glucose (mmol/l) >= 11.1 |
|  |  | 5.two-hour OGTT blood glucose (mmol/l) >= 11.1 |
|  |  | 6.Use of diabetes medication or insulin |
|  |  |  |
| **Hypertension [4]** | **NA** | 1. self-reported diagnosis of hypertension |
|  |  | 2. SBP≥140 mmHg and/or DBP≥90 mmHg |
|  |  | 3. the use of antihypertensive medication |
|  |  |  |
| **Marital Status** | **NA** | Divorced |
|  |  | Living with partner |
|  |  | Married |
|  |  | Never married |
|  |  | Separated |
|  |  | Widowed |

**Reference:**

[1] The role of hypertension in bone mineral density among males older than 50 years and postmenopausal females: evidence from the US National Health and Nutrition Examination Survey, 2005-2010 - PubMed. https://pubmed.ncbi.nlm.nih.gov/37397722/. Accessed 25 Oct 2023

[2] Rattan P, Penrice DD, Ahn JC, et al (2022) Inverse Association of Telomere Length With Liver Disease and Mortality in the US Population. Hepatol Commun 6:399–410. https://doi.org/10.1002/hep4.1803

[3] Serum selenium concentrations and risk of all-cause and heart disease mortality among individuals with type 2 diabetes - PubMed. https://pubmed.ncbi.nlm.nih.gov/34664061/. Accessed 25 Oct 2023

[4] Chen L, Zhang J, Zhou N, et al (2023) Association of different obesity patterns with hypertension in US male adults: a cross-sectional study. Sci Rep 13:10551. <https://doi.org/10.1038/s41598-023-37302-x>

**Supplementary Material 3. Overview of assessment characteristics for the study population across different NHANES cycles.**

|  | **2007-2008** | **2009-2010** | **2011-2012** | **2013-2014** | **2015-2016** | **2017-2018** | ***P*-value** |
| --- | --- | --- | --- | --- | --- | --- | --- |
| **Sociodemographic** |  |  |  |  |  |  |  |
| **Age; years, Mean (SE)** | 46.60 (0.62) | 47.29 (0.68) | 47.21 (0.77) | 47.47 (0.61) | 47.58 (0.65) | 46.40 (0.88) | 0.81 |
| **Sex n (%)** |  |  |  |  |  |  | 0.95 |
| Female | 942 (50.81) | 1031 (50.40) | 793 (49.61) | 937 (50.03) | 863 (51.10) | 681 (50.65) |  |
| Male | 923 (49.19) | 971 (49.60) | 860 (50.39) | 901 (49.97) | 858 (48.90) | 660 (49.35) |  |
|  |  |  |  |  |  |  |  |
| **Race n (%)** |  |  |  |  |  |  | 0.83 |
| Mexican American | 310 (7.73) | 364 (7.77) | 170 (6.93) | 243 (8.63) | 284 (7.83) | 193 (9.03) |  |
| Non-Hispanic White | 954 (72.22) | 1029 (71.96) | 711 (70.41) | 866 (68.94) | 637 (66.79) | 475 (65.56) |  |
| Non-Hispanic Black | 350 (10.67) | 323 (10.35) | 362 (10.39) | 334 (10.68) | 327 (10.27) | 298 (10.32) |  |
| Other Race | 251 (9.37) | 286 (9.92) | 410 (12.28) | 395 (11.75) | 473 (15.11) | 375 (15.09) |  |
|  |  |  |  |  |  |  |  |
| **Education Level n (%)** |  |  |  |  |  |  | 0.24 |
| Lower/higher secondary education | 993 (42.94) | 980 (39.90) | 685 (34.42) | 756 (35.60) | 760 (36.00) | 524 (35.40) |  |
| Some college or more | 872 (57.06) | 1022(60.10) | 968 (65.58) | 1082 (64.40) | 961 (64.00) | 817 (64.60) |  |
|  |  |  |  |  |  |  |  |
| **Ratio of family income to poverty n (%)** |  |  |  |  |  |  | 0.49 |
| low-income | 341 (12.60) | 423 (14.05) | 366 (14.92) | 395 (16.25) | 390 (14.45) | 205 (10.33) |  |
| middle-income | 1013 (47.11) | 1052 (49.89) | 862 (49.94) | 945 (48.81) | 923 (49.48) | 739 (49.03) |  |
| high-income | 511 (40.29) | 527 (36.06) | 425 (35.13) | 498 (34.94) | 408 (36.07) | 397 (40.63) |  |
|  |  |  |  |  |  |  |  |
| **Marital Status n (%)** |  |  |  |  |  |  | 0.41 |
| Divorced | 207 (9.78) | 224 (9.71) | 161 (10.29) | 201 (10.17) | 192 (9.42) | 168 (12.91) |  |
| Living with partner | 134 (7.48) | 168 (8.15) | 141 (8.25) | 125 (6.25) | 160 (10.01) | 132 (9.80) |  |
| Married | 1010 (57.31) | 1062 (57.13) | 812 (53.98) | 1009 (58.23) | 886 (55.66) | 677 (52.40) |  |
| Never married | 281 (17.04) | 327 (17.46) | 370 (20.85) | 336 (18.17) | 307 (17.79) | 244 (18.35) |  |
| Separated | 71 (2.65) | 55 (1.76) | 61 (1.81) | 52 (2.07) | 55 (2.05) | 46 (2.40) |  |
| Widowed | 162 (5.73) | 166 (5.79) | 108 (4.82) | 115 (5.13) | 121 (5.07) | 74 (4.15) |  |
|  |  |  |  |  |  |  |  |
| **Anthropometric** |  |  |  |  |  |  |  |
| **Body Mass Index; kg/m^2^, Mean (SE)** | 28.27 (0.15) | 28.67 (0.22) | 28.79 (0.28) | 29.29 (0.23) | 29.57 (0.32) | 29.37 (0.39) | **< 0.001** |
| **Waist circumference; cm, Mean (SE)** | 97.60 (0.45) | 98.46 (0.57) | 99.12 (0.69) | 99.98 (0.55) | 101.07 (0.86) | 99.73 (0.85) | **0.002** |
|  |  |  |  |  |  |  |  |
| **Healthy behavior factors** |  |  |  |  |  |  |  |
| **Dietary cholesterol intake; mg, Mean (SE)** | 308.31 (9.48) | 289.38 (6.79) | 286.64 (7.58) | 301.01 (5.87) | 291.64 (7.26) | 308.96 (7.41) | 0.16 |
|  |  |  |  |  |  |  |  |
| **Smoking status n (%)** |  |  |  |  |  |  | 0.64 |
| Never | 984 (53.98) | 1090 (54.98) | 937 (56.60) | 1037 (56.85) | 942 (53.56) | 786 (58.77) |  |
| Former | 491 (25.07) | 515 (26.40) | 382 (23.67) | 448 (24.69) | 440 (27.13) | 311 (25.10) |  |
| Now | 390 (20.95) | 397 (18.63) | 334 (19.73) | 353 (18.46) | 339 (19.31) | 244 (16.12) |  |
|  |  |  |  |  |  |  |  |
| **Alcohol intake n (%)** |  |  |  |  |  |  | **< 0.001** |
| Never | 245 (10.80) | 248 (10.25) | 203 (8.47) | 245 (11.47) | 273 (12.27) | 146 (7.04) |  |
| Former | 379 (16.23) | 350 (14.64) | 266 (13.85) | 312 (13.71) | 281 (13.97) | 0 (0.00) |  |
| Mild | 619 (37.40) | 668 (36.37) | 600 (39.48) | 640 (35.46) | 580 (36.26) | 613 (47.66) |  |
| Moderate | 258 (14.91) | 284 (16.60) | 243 (16.73) | 293 (19.02) | 265 (18.43) | 283 (21.25) |  |
| Heavy | 364 (20.66) | 452 (22.15) | 341 (21.47) | 348 (20.33) | 322 (19.07) | 299 (24.05) |  |
|  |  |  |  |  |  |  |  |
| **Moderate recreational activities n (%)** |  |  |  |  |  |  | 0.10 |
| Inactive | 1027 (46.87) | 1033 (44.54) | 773 (42.28) | 896 (48.39) | 893 (46.36) | 630 (39.30) |  |
| Active | 838 (53.13) | 969 (55.46) | 880 (57.72) | 942 (51.61) | 828 (53.64) | 711 (60.70) |  |
|  |  |  |  |  |  |  |  |
| **Biochemical data** |  |  |  |  |  |  |  |
| **Total cholesterol; mmol/L, Mean (SE)** | 5.03 (0.03) | 5.05 (0.04) | 5.00 (0.04) | 4.87 (0.03) | 4.92 (0.03) | 4.84 (0.05) | **< 0.001** |
| **Blood Urea Nitrogen; mmol/L, Mean (SE)** | 4.66 (0.06) | 4.79 (0.07) | 4.60 (0.05) | 4.74 (0.06) | 5.14 (0.09) | 5.19 (0.08) | **< 0.001** |
| **Serum uric acid; µmol/L, Mean (SE)** | 331.76 (3.05) | 327.80 (2.75) | 329.54 (3.78) | 325.58 (2.44) | 324.37 (2.23) | 321.90 (3.72) | 0.28 |
| **Creatinine; µmol/L, Mean (SE)** | 77.44 (0.73) | 78.48 (0.70) | 78.12 (0.77) | 79.59 (1.04) | 75.10 (0.75) | 76.01 (0.68) | **0.002** |
| **HDL-C; mmol/L, Mean (SE)** | 1.38 (0.02) | 1.40 (0.02) | 1.38 (0.02) | 1.39 (0.01) | 1.45 (0.02) | 1.42 (0.02) | 0.07 |
| **LDL-C; mmol/L, Mean (SE)** | 3.00 (0.02) | 3.02 (0.03) | 2.99 (0.03) | 2.89 (0.03) | 2.91 (0.02) | 2.88 (0.04) | **0.001** |
| **TG; mmol/L, Mean (SE)** | 1.41 (0.03) | 1.35 (0.03) | 1.39 (0.04) | 1.27 (0.03) | 1.24 (0.02) | 1.17 (0.02) | **< 0.001** |
| **Insulin; μU/mL, Mean (SE)** | 11.52 (0.43) | 13.40 (0.26) | 13.06 (0.43) | 12.04 (0.44) | 13.08 (0.38) | 12.70 (0.60) | **0.005** |
| **Fasting glucose; mmol/L, Mean (SE)** | 5.83 (0.04) | 5.73 (0.06) | 5.80 (0.07) | 5.83 (0.05) | 6.07 (0.04) | 6.04 (0.07) | **< 0.001** |
| **HOMA-IR; units, Mean (SE)** | 3.13 (0.14) | 3.57 (0.10) | 3.57 (0.17) | 3.42 (0.16) | 3.90 (0.14) | 3.70 (0.22) | **0.01** |
| **HbA1c; %, Mean (SE)** | 5.55 (0.03) | 5.60 (0.03) | 5.61 (0.04) | 5.62 (0.03) | 5.68 (0.03) | 5.59 (0.04) | 0.12 |
| **NHHR; units, Mean (SE)** | 2.87 (0.04) | 2.86 (0.05) | 2.83 (0.05) | 2.74 (0.04) | 2.71 (0.04) | 2.63 (0.05) | **< 0.001** |
|  |  |  |  |  |  |  |  |
| **Chronic disease factors** |  |  |  |  |  |  |  |
| **Hypertension n (%)** |  |  |  |  |  |  | 0.71 |
| No | 1060 (63.96) | 1183 (64.57) | 974 (61.18) | 1067 (61.26) | 974 (62.16) | 780 (63.30) |  |
| Yes | 805 (36.04) | 819 (35.43) | 679 (38.82) | 771 (38.74) | 747 (37.84) | 561 (36.70) |  |
|  |  |  |  |  |  |  |  |
| **Stroke n (%)** |  |  |  |  |  |  | 0.50 |
| No | 1783 (96.97) | 1926 (96.93) | 1583 (96.51) | 1776 (96.83) | 1656 (97.29) | 1298 (97.99) |  |
| Yes | 82 (3.03) | 76 (3.07) | 70 (3.49) | 62 (3.17) | 65 (2.71) | 43 (2.01) |  |
|  |  |  |  |  |  |  |  |
| **T2DM n (%)** |  |  |  |  |  |  | 0.14 |
| No | 1462 (85.39) | 1608 (84.61) | 1315 (85.55) | 1492 (84.52) | 1305 (81.35) | 1078 (86.41) |  |
| Yes | 403 (14.61) | 394 (15.39) | 338 (14.45) | 346 (15.48) | 416 (18.65) | 263 (13.59) |  |

**Notes:** All values are presented as number (%) for categorical variables, assessed via weighted chi-square tests, or mean (standard error) for continuous variables, assessed via weighted Student’s t-tests.

**Abbreviations**: **NHHR**: non-high-density lipoprotein cholesterol to high-density lipoprotein cholesterol ratio; **T2DM:** type II diabetes mellitus; **TG**: Triglyceride; **HDL-C**: high-density lipoprotein cholesterol; **LDL-C**: low-density lipoprotein cholesterol; **HOMA-IR:** Homeostatic Model Assessment of Insulin Resistance; **HbA1c:** hemoglobin A1c; **SE:** standard error.
